# Application of concentrated growth factor to autotransplantation with inflammation in recipient area

**DOI:** 10.1186/s12903-021-01915-3

**Published:** 2021-10-30

**Authors:** Dilinuer Keranmu, Ailimaierdan Ainiwaer, Nijiati Nuermuhanmode, Wang Ling

**Affiliations:** Outpatient Department of Oral Surgery, The First Affiliated Hospital of Xinjiang Medical University(Affiliated Stomatological Hospital), Research Institute of Stomatology of Xinjiang Uygur Autonomous Region, No. 393, Xinyi Road, Xinshi District, Urumqi, Xinjiang, 830054 China

**Keywords:** Autogenous tooth transplantation, Mature impacted tooth, Inflamed recipient site, Concentrated growth factor, 3D replica model

## Abstract

**Objective:**

The purpose of this study was to apply concentrated growth factor (CGF) to the transplanted area with inflammation, to observe the clinical effects of CGF on the inflammation area assisted by 3D printing technology.

**Methods:**

A total of 52 compromised mandibular first or second molar with chronic periapical lesions were transplanted with mature third molars. The patients were divided into CGF group (n = 26) and control group (n = 26) and transplanted into fresh extraction sockets with or without CGF. All the patients underwent clinical and radiographic examinations during the follow-up.

**Results:**

Average surgery and extra-oral time were 39 min (± 7.8) and 42 s (± 10.2). The success rates of CGF group and control group were 100% and 92.3% respectively. Most of the periapical lesions in CGF group healed completely within 3 months, which was significantly faster than control group. The initial stability of CGF group was better than control group immediately after operation, and the degree of pain in CGF group was lower than control group on the 1st and 3rd day after operation.

**Conclusions:**

The application of CGF in recipient site with chronic periapical lesions can accelerate the regeneration of alveolar bone and the healing of inflammation, greatly shorten the healing period. Meanwhile, CGF help to reduce postoperative pain and reaction at the early stage of healing and increase the success rate of autogenous tooth transplantation (ATT). Additionally, the use of 3D printing model can greatly reduce the extra-oral time of donor teeth.

## Introduction

Loss of molars have various causes, such as dental caries, trauma, periodontal disease, etc. [1]. Tooth loss not only affects the masticatory function and facial aesthetics of patients, but also has negative impact on mental health and quality of life. At present, there are three major methods to restore missing tooth, fixed partial denture, removable partial denture and implant. In addition, the autogenous tooth transplantation (ATT) has become an effective and acceptable treatment option for missing tooth. The history of tooth transplantation can be traced back to the 1950s [2]. ATT refers to the transplantation of physically intact tooth without biological function, such as impacted, dislocated or ectopic tooth, from one position to another to support the masticatory function of the affected tooth. ATT is most commonly used in the third molar to replace the damaged first or second molar [3]. ATT requires a complete extraction of the donor tooth without any damage or cracks, and the periodontal ligament (PDL) on the root surface needs to be preserved, which is a critical problem affecting the prognosis of the donor tooth [4].

A systematic review indicated that the overall success rate of ATT was 89.68%, which is comparable to the 10-year of a dental implant [5]. Such treatment not only provides improved aesthetics, arch forms, PDL, proprioception [6], but also greatly induces the regeneration of alveolar bone in three dimensions and the formation of gingival papilla. Besides, orthodontic movement of the transplanted tooth can be carried out without the risk of immune rejection [1, 7]. The short-term treatment period and lower cost made ATT an alternative method.

It is known that the PDL consists of fifibrous connective tissue containing cells, nerves, and blood vessels and plays a key role in regulating the bone remodelling that occurs during tooth movement [8]. Studies have shown that the shorter the extra-oral time, the less likely the PDL was to be damaged [9]. Compared with traditional methods, oral cone beam computed tomography (CBCT) guided method is extremely helpful during the preparation of the recipient socket as it minimized the trauma to the PDL and Hertwig’s root sheath, reduced the extra-oral time of the donor tooth, and improved the predictability of surgery [10]. Increasingly studies have shown that 3D printing model based on CBCT data is an economical and effective method, which can reduce the operation time and the potential iatrogenic damage to the donor tooth [11]. Therefore, computer-aided design (CAD) and 3D printing technology were applied in our study to improve the success rate of ATT.

Third molars, premolars, ectopic and supernumerary teeth can be used as donors. Success rate of the fully developed premolars was higher than 90% [12]. Zufía, et al. [13] carried out a study using the third molar to replace the mandibular second molar suffered vertically fractured and reaped the satisfactory results. However, the presence of inflammation in the recipient site restricted the selection of indications and the development of ATT to a certain extent.

As the third generation of concentrated platelet, CGF was first proposed by Sacco in 2006. CGF is made from autologous venous blood with no addition of any biological agents, non-toxic, no immunogenicity, and separated by special centrifugation. CGF has strong tissue regeneration ability and biodiversity, stable fibrin matrix, high tensile strength, and a large quantity of osteoblasts. Collectively, CGF is a powerful biological scaffold and growth factor library [14]. CGF has been extensively used in various situations, ranging from the fifilling of extraction sockets [15] to the fifilling of a cavity after cystectomy [16], implant surgery [17], sinus augmentation procedures [18], simple GBR procedures or as a membrane support in recession coverage [19]. Further, CGF is considered to relieve postoperative pain and swelling, and reduce the occurrence of alveolar osteitis [20]. Nevertheless, there are few studies on ATT in the case of inflammation in the periapical, and the application of CGF in ATT has not been reported yet.

Therefore,the purpose of this study was to apply CGF to the transplanted area of chronic periapical lesions with the help of 3D printing technology, to observe its effects on the inflammation of the recipient area, the osteogenesis of periroot bone defects, and explore the clinical effects of CGF in the treatment of inflammatory ATT.

## Materials and methods

Fifty-two patients who underwent ATT in department of oral surgery in our hospital from January 2018 to December 2020 were enrolled in this study. All the donor teeth were third molars with closed apices, and chronic periapical lesions exists in the recipient area. Patients were informed about the potential benefits and risks of surgery, as well as alternative treatment options, and volunteered to participate in and signed an informed consent. The protocol of this study was consistent with the ethical guidelines of the Declaration of Helsinki.

### Subjects

Patients included in this study met the following criteria:

1. First or second molar diagnosed as chronic periapical lesions and need to be extracted.
2. Mature third molars with no function or need to be extracted for treatment.
3. The shape and size of the third molars was close to the extracted tooth, with a suitable space in the recipient area.
4. The buccal or lingual wall defect in the receiving area was assessed to be less than 1/3 of the alveolar bone wall (including height and width).
5. Good oral hygiene and compliance.

Patients with the following conditions were excluded:

1. Root variation, the donor tooth could not be extracted completely.
2. Poor occlusal relationship.
3. With surgical contraindications, systemic and local factors that affect the healing of the wound.

### Preoperative work-up

**Fig. 1:**
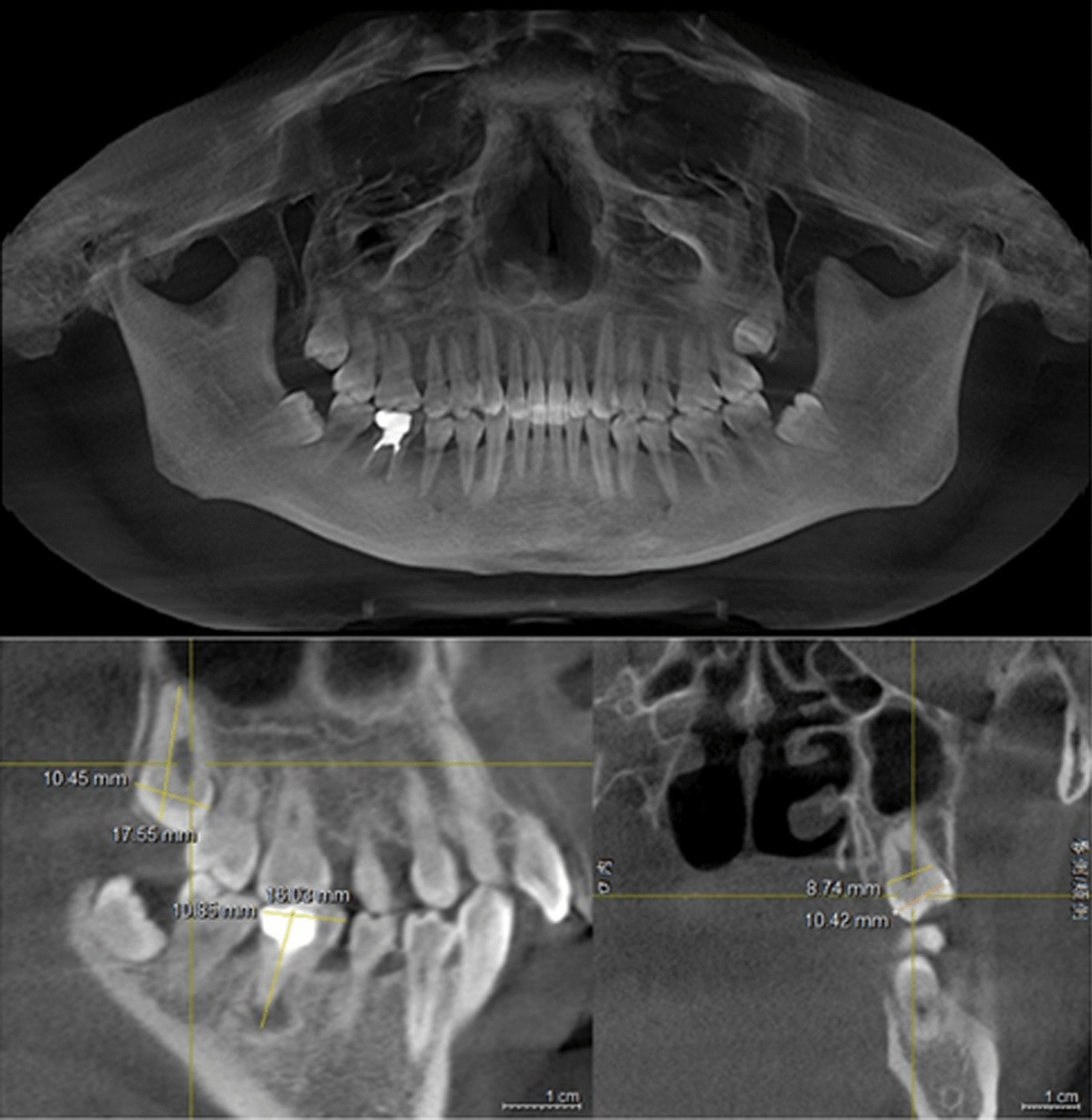
The measurements of the CBCT for preoperative evaluation. sagittal view: the measurements of right mandibular first molar (#46) and right maxillary third molar (#18); coronal view: the measurements of mesial/proximal and distal width of crown and root neck (#18)

Preoperative examination including general conditions, routine blood tests, oral hygiene, occlusal relationship, etc. When necessary, supragingival scaling was performed. CBCT was taken to evaluate the mesial/proximal and distal width of crown and root neck of the donor tooth (Fig. 1). Meanwhile, the degree of root development, number and curvature were examined to evaluate whether it could be extracted successfully and completely. Besides, the position between the recipient area and the inferior alveolar nerve was taken into consideration.

**Fig. 2:**
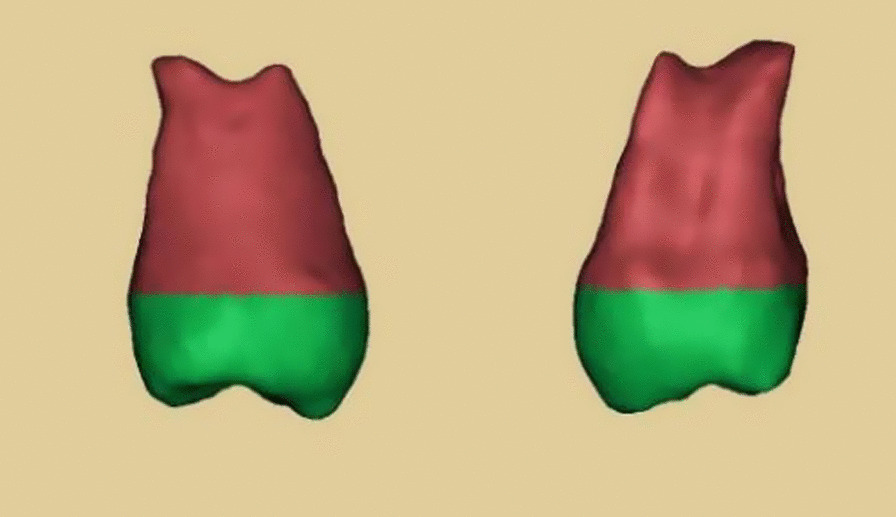
The three-dimensional (3D) image of the donor tooth

The data obtained by the CBCT was converted into DICOM format, and then imported into MIMICS software and 3D printer to replicate and print the resin tooth model (Fig. 2). Acrylic models were sterilized by ethylene oxide before surgery, aseptically packed and ready for use.

**Fig. 3:**
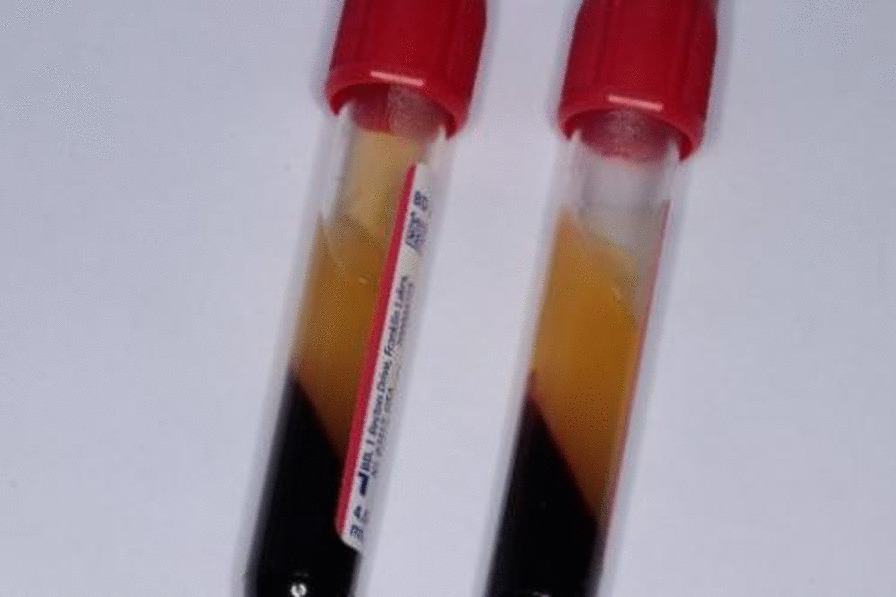
The CGF obtained by centrifugation

Venous blood samples were collected from each patient in the CGF group and injected into two sterile 10-mL 2-blood tubes (vacuum negative pressure inside, tube wall coated with silica particles, and without anticoagulant) respectively. The tubes were placed symmetrically in a Medifuge centrifuge (Silfradent, Italy) and CGF was prepared according to the specified procedure. As described by Bozkurt et al., after centrifugation, the CGF layer, which was the second of the three layers, was separated with sterile scissor (Fig. 3).

### Surgical procedure

All operations were performed by the same experienced oral surgeon. The oral was cleaned with compound chlorhexidine, and the injection site of anesthesia and operation area were disinfected with iodophor cotton balls. After local anesthesia was performed with 2% lignocaine in 1: 200,000 dilution adrenaline, the compromised molar was extracted atraumatically. The periapical lesion was curetted by using bone curettes and the alveolar fossa was preliminarily prepared. All operations were performed under irrigation with sufficient sterile saline, to provide clear vision and reduce the thermal damage to the extraction socket. The 3D model of the donor tooth was used to guide the modification of the alveolar socket until it was completely in place. In the process of preparation, the model can be put in and taken out for several times, the direction and angle could be changed arbitrarily without special protection and fully moisturizing. After minimally invasive extraction, the impacted tooth was immediately transplanted into the recipient socket to check whether they reached the ideal position. When further adjustment was needed, the donor tooth was placed in the sterile saline.

In the CGF group, the "gauze method" was used to make the “CGF membrane” by pressurizing and absorbing the liquid. Before transplantation, the CGF membrane with certain elasticity and adhesion was placed at the bottom of the extraction socket in the recipient area, so that the CGF membrane wrapped the root and completely covered the inflammatory area. In the control group, the donor tooth was simply transplanted after the inflammatory tissue was curetted with saline irrigation. The donor tooth was put in place by using finger pressing without touching the root.

**Fig. 4:**
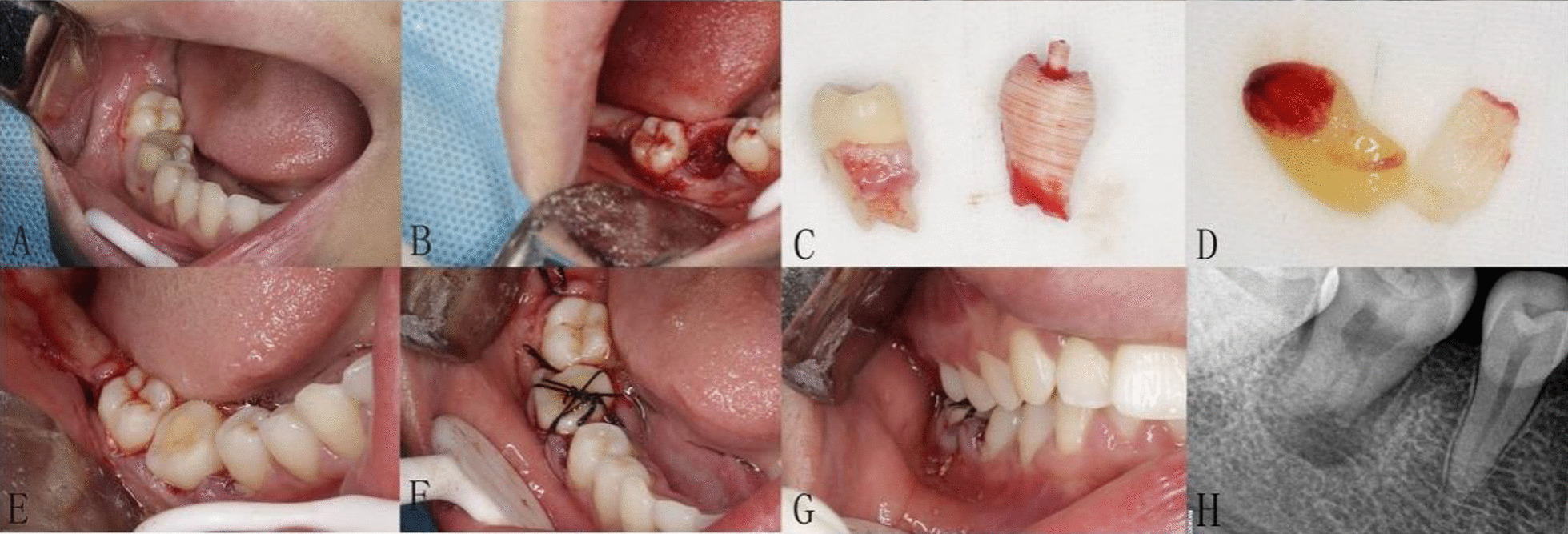
Surgical proceduce of right maxillary third molar transplanted into fresh socket of right mandibular first molar. **a** Compromised first molar tooth; **b** fresh socket of the first molar tooth after extraction; **c** the 3D replica was almost the same of the donor maxillary third tooth; **d** the CGF membrane made by pressurizing and absorbing the liquid; **e** try-in of the donor tooth; **f, g** suturing the flap and fixed the donor tooth; **h** postoperative peri-apical radiograph was taken immediately after autotransplantation

After transplantation, teeth with good initial stability were fixed with "8" suture for 1 week, and the teeth with poor initial stability were fixed with fiber-glass band for 4 weeks. The brief surgical procedure of the ATT was showed in Fig. 4.

### Postoperative examination and treatment

Visual analogue scale (VAS) was used to measure the degree of postoperative pain on day 1, 3, and 7. All patients were treated with antibiotics 1 day before operation for 5 days and underwent mouth rinsing for 1 week. The grade of mobility of the transplanted tooth was examined at 1 week and 4 weeks after the removal of sutures and fiber-band fixation, respectively. Root canal treatment (RCT) was carried out when the grade of mobility was less than or equal to grade I, if it was more than grade I, the time of RCT was postponed. The periodontal assessments were performed at every visit, including bleeding on probing, pocket probing depth, clinical attachment loss, pain to percussion and mobility grade. The periapical radiograph was taken immediately, 2 weeks, 1, 3, 6, 12 and 24 months after operation.

The success criteria of our study were determined according to the description of Andreasen et al. [9] In terms of clinical examination (1) physiologic mobility; (2) no percussion pain; (3) probing depths ≤ 3 mm; (4) no signs of inflammation; (5) normal masticatory function. In terms of X-rays (1) normal space of the parodontium; (2) no progressive resorption of the root; (3) the presence of the lamina dura. ATT was considered as a failure when the inflammation of recipient area still exists or when the transplanted tooth appeared clinically unhealthy with persistent mobility (grade III), ankylosis, and progressive root or marginal bone resorption.

## Results

The follow-up period ranged from 18 to 36 months, with an average of 26 months. One patient in the CGF group successfully completed RCT and immigrated abroad in the first year after operation without further examination and excluded in the further evaluation. A total of 51 mature third molars (22 males and 29 females, mean age 32.6 ± 6.16, range from 21 to 46 years) were evaluated (Table 1). All the recipient areas located in fresh first or second mandibular molar, and there was no significant difference in the diameter of periapical lesions between the two groups. Table 2 shows the distribution of the transplanted teeth and recipient sites. The average surgery and extra-oral time were 39 min (± 7.8) and 42 s (± 10.2).

**Table 1:** Number and distribution of transplanted teeth by gender, age at transplantation, duration of operation, and extra-oral time of donor teeth

	Basic information of patients		Total
	CGF group	Control group	
Case number	26	26	52
Gender			
Male	12	11	23
Female	14	15	29
Age, years (range)	32.4 (22–45)	32.9 (24–46)	32.63 (22–46)
Operation time, min (range)	38.6 (30–55)	40 (30–55)	39.3 (30–55)
Extra-oral time of donor tooth, second (range)	44.8 (30–60)	38.8 (30–55)	41.8 (30–60)

**Table 2:** Distribution of the transplanted teeth and recipient sites

Transplanted third molar	Recipient sites				Sum
	36	37	46	47	
18	5	0	0	5	10
28	5	0	3	1	9
38	8	3	4	3	18
48	2	6	3	4	15
Sum	20	9	10	13	52

The initial stability of CGF group was better than control group immediately after operation. (*P* < 0.05)(Table 3). The degree of pain in CGF group and control group was significantly higher on the first day after operation and decreased gradually within 7 days. The VAS score of CGF group was lower than control group on the 1st and 3rd day after operation (*P* < 0.05)(Table 4).

**Table 3:** The stability of transplanted teeth immediate and postoperative 1 and 4 weeks after transplantation

Group	Stability		
	After operation	1 week	4 weeks
CGF group	1.19 ± 0.491	0.77 ± 0.514	0.12 ± 0.326
Control group	1.81 ± 0.491	0.88 ± 0.516	0.27 ± 0.604
T-test	4.515	0.808	1.143
*P* value	0.001	0.423	0.258

**Table 4:** Degree of pain experienced after surgery assessed by VAS value at day 1, 3, 7

Group	Visual analogue scale (VAS)		
	1 day	3 days	7 days
CGF group	4.750 ± 0.7778	3.385 ± 0.8283	1.673 ± 0.5281
Control group	5.538 ± 0.8823	4.019 ± 0.8060	1.981 ± 0.6555
T-test	3.418	2.800	1.864
*P* value	0.001	0.007	0.068

**Fig. 5:**
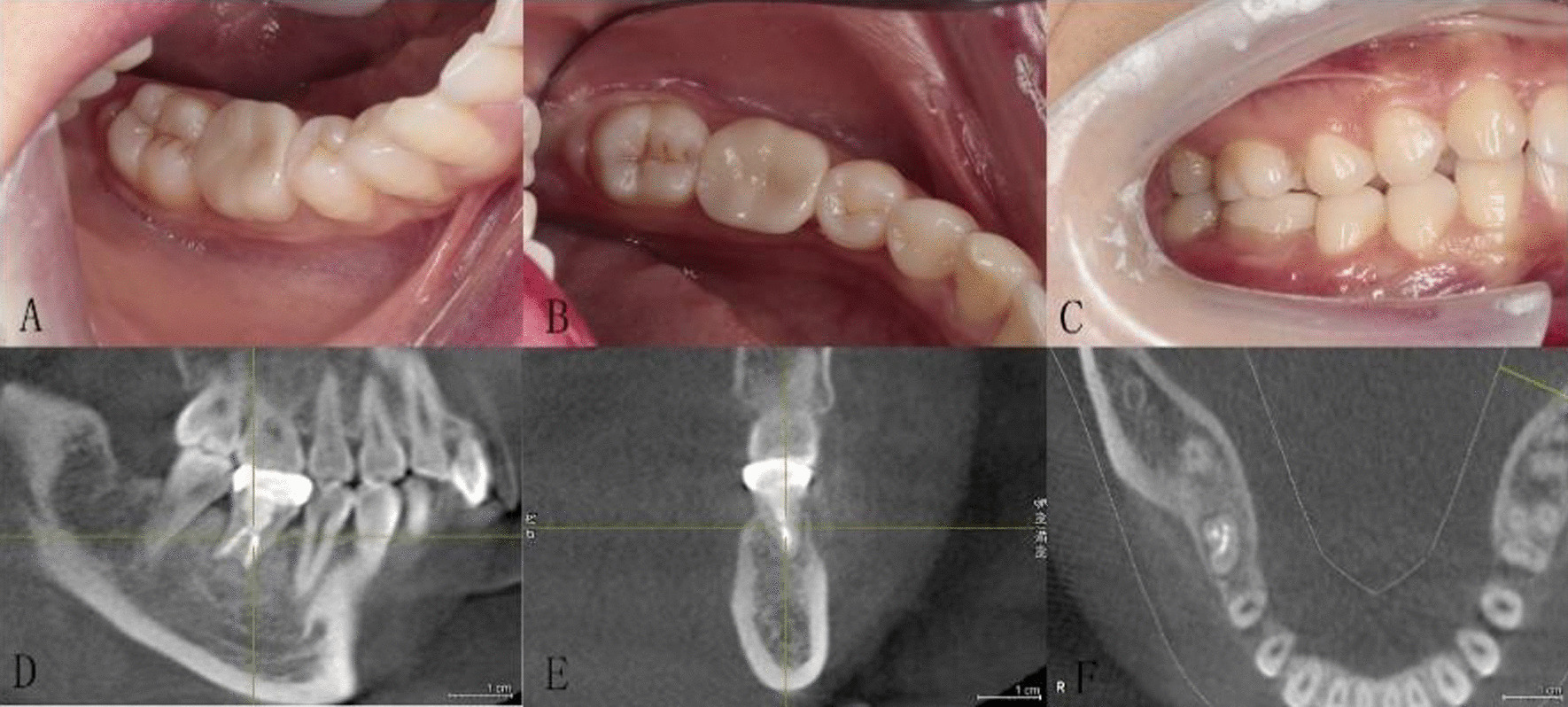
**a–f** Intraoral and CBCT images were taken 2 years after operation. Transplanted teeth in CGF group showed acceptable chewing function, and there was no pathological radiation, abnormal mobility, periodontal pocket, root resorption, ankylosis and any other adverse events

**Fig. 6:**
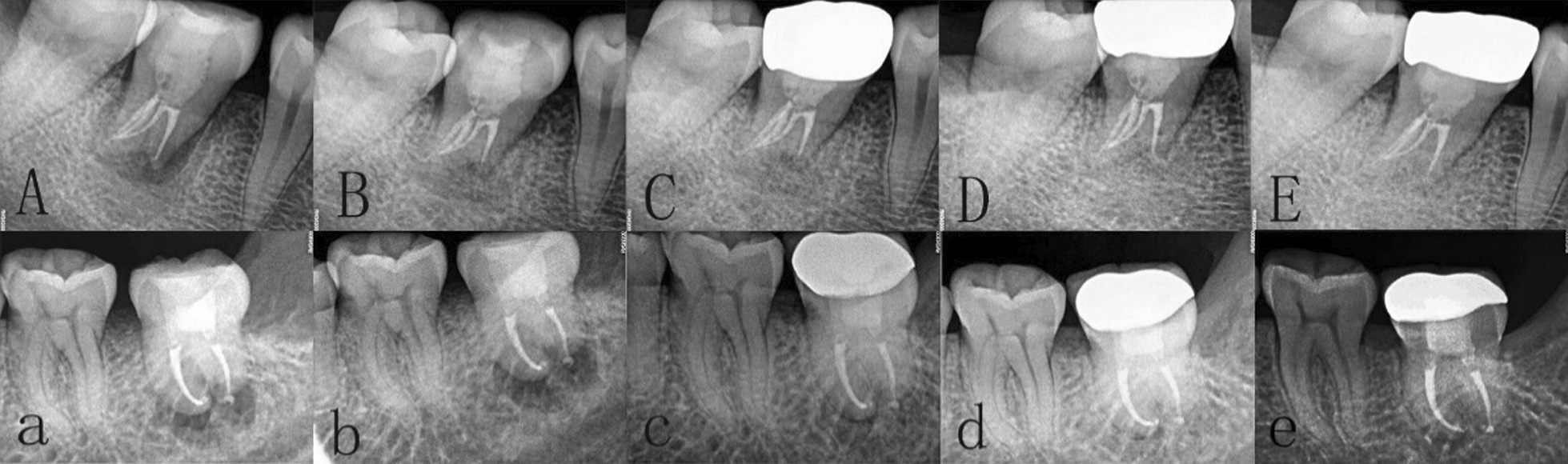
Periapical X-ray examination was performed at 1, 3, 6, 12 and 24 months after operation. Above: **A–E** is from CGF group. Below: **a–e** is from control group

In the CGF group, periapical radiography showed that periapical lesions of 23 out of the 26 cases (88%) healed completely compared with normal alveolar bone within 3 months, that is, 23 cases met the success criteria of our study, and the other 3 cases (22%) within 6 months. In the control group, 9 cases (35%) showed complete healing within 3 months, and 17 cases (65%) within 6 months (Tables 5, 6). During the 2-year follow-up, all the patients in CGF group showed acceptable chewing function, and there was no pathological radiation, abnormal mobility, periodontal pocket, root resorption, ankylosis and any other adverse events (Figs. 5, 6). All of 25 transplanted teeth reached the standard of success, resulting in a 100% success rate in CGF group. In the control group, some probing depths of the transplanted teeth were deeper than 3 mm at 4 weeks or 3 months temporarily. 2 cases were failed in control group, with a total success rate of 92.3%. The success rate of CGF group was higher than control group (*P* > 0.05).(Table 7). The absolute values of each clinical and radiological parameter evaluated after 2-year follow-up are shown in Table 8.

**Table 5:** Bone healing of CGF group and control group at 3 and 6 months after operation

	CGF group		Control group	
	After 3 months	After 6 months	After 3 months	After 6 months
Case, n	26	26	26	26
Healing, n (%)	23 (88%)	26 (100%)	9 (35%)	24 (92%)
Mal-healing, n (%)	3 (12%)	0 (0%)	17 (65%)	2 (8%)
Healing rate (%)	88%	100%	35%	92%

**Table 6:** Bone healing of CGF group and control group at 3 and 6 months after operation

Group	Diameter of periapical lesion	Healing of alveolar bone	
	(in millimeter)	After 3 months	After 6 months
CGF group	15.69 ± 4.416	1.12 ± 0.326	1.00 ± 0.00
Control group	15.62 ± 4.891	1.65 ± 0.485	1.08 ± 0.272
T-test	0.060	4.698	1.443
*P* value	0.953	0.01	0.155

Remarks: When the data were recorded, the diameter of periapical lesion was expressed as the maximum diameter (length*width). 1 represented bone healing and 2 represented no significant change in periapical lesions

**Table 7:** Results of 2 years follow up in CGF group and control group

	Outcome		
	CGF group	Control group	Total
Case, n	26	26	52
Missed follow-up, n (%)	1 (3.8%)	0 (0%)	1 (1.9%)
Fail, n (%)	0 (0%)	2 (7.6%)	2 (3.9%)
Success rate	100%	92.3%	96.1%

**Table 8:** Absolute numbers for each clinical and radiographic parameter assessed after 2-year follow-up

	Present	Absent
Symptomatology	2	49
Bleeding on probing	1	50
Probing depths > 3 mm	1	50
Clinical attachment loss	0	51
Mobility ≥ grade III	1	50
Pain in percussion	1	50
Signs of inflammation	1	50
Abnormal masticatory function	0	51
Abnormal space of the parodontium	1	51
Progressive resorption of the root	1	50

## Discussion

Herein, we evaluated the impact of CGF on the healing of inflammation and osteogenesis in the transplanted area via radiographic and clinical evaluations. This approach confirmed that CGF application is safe and more effective than natural healing as a means of accelerating the regeneration of new bone and the healing of inflammation, and greatly shorten the healing period of ATT.

An increasing number of studies have shown that ATT is an alternative method for the repair of missing teeth, which can provide immediate and permanent restoration [1]. The success rate of ATT was affected by several factors, such as the degree of root development, age, overall periodontal condition, extra-oral time of the donor tooth and the volume of alveolar bone in the recipient area [21]. ATT can achieve ideal effect when the root of donor tooth was developed to 1/2–3/4 of its length. While tooth with unclosed apical has higher success rate and a lower possibility of complications [22], the third molar with complete root formation, even the supernumerary tooth, can also be used as donor. Later treatments such as RCT, resin filling and crown restoration can also achieve good functional and aesthetic effects without affecting the success rate of ATT [4].

Molars are easy to loss early because of large number of pits and fissures, early eruption, caries, cracks or other diseases. Third molars with completely formed roots are similar in shape, size and length to the first and second molars, so in our study all of the recipient areas were mandibular molars in our study. However, the probability of re-vascularization in mature molars is almost impossible due to the closure of apical foramen, but the PDL can be reconstructed [23]. The healing of PDL depends on the number and viability of periodontal ligament stem cells (PDLSCs) attached to the root surface [9]. Nevertheless, when the recipient socket and donor tooth does not match perfectly, the socket and tooth need further trimming, which extends the extra-oral time. In order to maintain the viability of PDLSCs, minimal manipulation is necessary to save the Hertwig’s root sheath, and extra-oral time of donor tooth should be limited within 5 min [24]. Verweij et al. [10] reported a mean extra-oral time of 7.6 min with the absence of 3D printing model, ranging from almost 0 to 25 min. However, in the present study, all the donor teeth were transplanted into the recipient site within 60 s after extraction, and such a short time was due to the auxiliary application of 3D printing model. Consistent with the reports of Cousley [11] and Ashkenazi [25], we found that the 3D model can reduce the frequency of tooth fitting to a certain extent, reduce the iatrogenic damage to the PDL and shorten the extra-oral time. Meanwhile, by means of 3D model, the alveolar socket can be prepared accurately, so that the operation is more predictable.

The main difference of this study from the other similarly published ones is that all recipient sites with chronic periapical lesions in CGF group were treated with CGF. CGF has been recommended to replace bone graft and membrane to enhance bone regeneration and shorten the whole treatment period [26]. When the conventional non-surgical methods cannot achieve the purpose of treatment, apical surgery is the preferred choice for patients with persistent periapical periodontitis [27]. Smaller defects can be healed in about one year, while larger defects can take more than two years. Nevertheless, in one case report, two patients with extensive periapical lesions were treated with CGF, after 6 months of follow-up, ideal bone healing was found [26]. Feifei et al. [15] detected that CGF can be used as a socket filling material following posterior tooth extraction in order to achieve ridge preservation over a 3–3.5 months observation period. Huang et al. [28] evaluated the effect of CGF in alveolar cleft. They performed CBCT evaluation and concluded that the bone resorption rate and the bone density improvement with better results in CGF group than in ADM (acellular dermal matrix) group. Moreover, CGF was used as a substitute for bone graft in sinus augmentation and the results showed that the healing was reduced to half of the average healing time [29]. Besides, Some scholars attempted to perform ATT in the inflamed recipient area, 6 months after surgery, the periapical bone of 12 patients was completely healed compared with the healthy jaw bone [30]. Whereas, different from aforementioned, according to the radiographical results, we found most of the cases in the CGF group demonstrated complete healing of the periapical lesions within 3 months, the overall success rate of CGF group was 100%, while the inflammatory healing speed and bone regeneration in control group were significantly slower. The 2-year follow-up has affirmed the encouraging effect of CGF which has shortened the healing time of periapical lesions to 3 months. It can be seen that the application of CGF was conducive to inflammatory healing and bone regeneration.

CGF mainly consists of two parts, a three-dimensional network scaffold composed of fibrinogen molecules of varying thickness and various types of cells and growth factors "inlaid" in it. With large porosity and good elasticity, the fibrin three-dimensional grid can not only be used as a "storeroom" for various cells and growth factors, but also can be released in tissues slowly with the degradation of collagen [31]. Studies have confirmed that CGF contains at least 16 key cell growth factors, such as platelet-derived growth factor (PDGF), transfer growth factor-β (TGF-β), insulin-like growth factor (IGF-1), epidermal growth factor (EGF), bone morphogenetic proteins (BMPs), vascular endothelial growth factor (VEGF) and fibroblast growth factor (FGF), etc. Moreover, it can be used as a growth scaffold for osteoblasts, fibroblasts, endothelial cells to guide the deposition and internal growth of new bone. Different from PRF, CGF is centrifuged at 2400–2700 r min^−1^, which maximizes the release of platelet α particles, increase the collision rate between platelets, so as to obtain higher concentration and more abundant types of growth factors. The internal growth factors and fibrin matrix can promote the rehabilitation of soft/hard tissues and wound healing.

It is known that the process of wound healing includes three consecutives but overlapping stages, biochemical activity, cell activity, and cell reaction phase [32]. The Hagemann factor in the serum initiates and promotes the biochemical activity phase, which leads to the beginning of cellular activity phase. The autologous CGF, thanks to the abundant presence of growth factors, represents a valid aid for the acceleration of the repair processes and the regeneration of hard and soft tissues, in oral surgery [33]. Among which, PDGF is the earliest growth factor that appears on the wound surface, which can facilitate the chemotaxis and proliferation of cells, increases the synthesis ability of collagen, and stimulate the rapid growth of granulation tissue [34]. TGF-β, as an important regulatory factor in the process of bone formation and remodeling, controls inflammation through synthetic fibrous connective tissue and local vascular proliferation, and also induces regeneration of alveolar bone [33]. IGF-1 can enhance the migration, division, proliferation and chemotaxis of related cells, and plays an important role in the growth, remodeling and repair of bone. The BMPs act as growth and differentiation factors, and as chemotactic agents. They stimulate angiogenesis, migration, proliferation, and differentiation of stem cells from the surrounding mesenchymal tissues into bone-forming cells in an area of injury [35]. Furthermore, VEGF is involved in the progression of periapical periodontitis, and its expression in the PDL and bone tissues increases with the progression of inflammation. VEGF can produce more bone morphogenetic proteins, accelerate the re-vascularization of damaged tissues, promote the differentiation and growth of osteoblasts and osteoclasts. Furthermore, VEGF in CGF was 1.5 times of PRF [36], and thus CGF obtained a larger and denser fibrin clot with higher tensile strength and adhesion. Compared with PRP and PRF, CGF can release growth factors within 13 days at least [37], the actuation duration is prolonged, which indicates that it has better repair and regeneration ability. In addition, CGF fibrous scaffold contains a considerable number of leukocytes, which enhances its anti-infection ability.

In order to obtain satisfactory therapeutic effect, postoperative pain is often the common concern of clinicians and patients. We evaluated the postoperative pain and found that the VAS score of the two groups was the highest on the first day after transplantation. What is more, the VAS score of CGF group was significantly lower than control group on the 1st and 3rd day, which proved CGF could relieve postoperative pain. This result is consistent with the findings of Akcan [19], who also pointed out that platelet concentrate can improve the postoperative adverse reactions.

Fixation methods after transplantation depends on the initial stability of the transplanted teeth. Maintaining a good initial stability is essential for the healing of PDL and osseointegration [38]. Compared with control group, the initial stability of CGF group was significantly improved immediately after the operation. We considered this may be because CGF was placed at the bottom of the alveolar socket and filled in the recipient site, so that the alveolar socket can wrap around the root well and reduce the gap between root and alveoli fossa. The decrease and subsequent increase in the stability of control group were attributed to remodeling during bone healing. It reflects that CGF may affect the initial stability of transplant by accelerating the osseointegration process [39]. In addition, CGF contains high concentration of growth factors, platelets and fibrin, which has great tensile strength, agglutination and adhesion, and can provide stability for the transplant to a certain extent. At the same time, CGF can prevent direct contact between root and alveolar bone to reduce the adverse effects caused by contact and may reduce the occurrence of ankylosis. In terms of the fixed time, we found the formation of periodontal pockets may be related to long-term fixation. Consequently, long term rigid fixation is not recommended.

All the transplanted teeth in our study were required to receive RCT within 2–4 weeks after transplantation when they showed physiological mobility. Specifically, when the mobility of the teeth with "8" suture was less than or equal to grade I, RCT should be carried out within 2 weeks, while resin-bonded teeth should be carried out at 4 weeks. The time of RCT was delayed if transplanted teeth have abnormal mobility. However, the speed of osteogenesis and prognosis of the transplanted teeth which completed RCT within 2 weeks were better than those about 4 weeks. While some scholars have proposed that extra-oral RCT and ATT should be carried out at the same time, this not only increases the extra-oral time and affects the viability of PDLSCs, but also may result incomplete removal of microorganisms in the root canal [40]. Therefore, for the tooth with closed apical foramen, RCT should be performed within 2–4 weeks after operation to prevent complications such as apical inflammation and root resorption, which may cause the failure of ATT. In this regard, it is appropriate to consider some authors suggesting that root resorption phenomena are counteracted by a subsequent phase of bone remodeling and new apposition without infectious events [41].

It is worth noting that after RCT, due to lack of blood supply, the fragility of the tooth will increase, and will prone to fracture after long-term use, and the masticatory function cannot be performed when the occlusal relationship is not resumed well. For these reasons, all patients in our study were required to undergo postoperative crown restoration, and all of them achieved good masticatory function during the follow-up with high satisfaction. Therefore, crown restoration after RCT was recommended to obtain good masticatory function and aesthetics.

Many other factors also affect the success rate of ATT, such as age and gender. Yoshino et al. [42] highlighted that the younger the patients, the higher the success rate of ATT. This can be explained by the fact that the older the age is, the higher the incidence of caries and periodontitis and the slower the metabolism. In addition, the bone mineral density (BMD) will also change, which make it difficult to extract and prepare the recipient site. The two patients who failed in this study were all male over 40 years old, one of which was due to the probing depth at the distal-buccal/lingual site was deeper than 3 mm, and the other patient did not receive RCT on schedule and external root resorption was found at 2-year follow-up. Therefore, it was suggested that patients underwent ATT should receive RCT on time and continue to maintain postoperative oral hygiene to improve the prognosis of ATT.

There are certain limitations to the present study, including relatively small sample size and short follow-up duration. Although characterized by low power of the evidence, the results of this study are encouraging and provide clinicians with a viable alternative to address the great challenge of inflammation in transplantation area. Meanwhile, it will be necessary to increase the patient sample and the follow up period to be able to confirm such preliminary results, evaluate the potential of growth factors and expand the indications of ATT.

## Conclusion

All the results obtained are in agreement to show CGF is a valid aid in speeding up the processes of bone regeneration. Specifically, the application of CGF in transplantation area with chronic periapical lesions can accelerate the formation of new bone and the healing of inflammation, greatly shorten the healing period. Meanwhile, CGF help to reduce postoperative pain at the early stage of healing and postoperative reaction, and increase the success rate of ATT. Certainly, further studies are needed to analyze in more detail the CGF and their performance.
